# Reviewed article: Cruz GL, Andrade GC, Rauber F, Levy RB, Louzada MLC. Application of the Nova food classification in the 2017-2018 Family Budget Survey: a strategy for monitoring adherence to the recommendations of the Food Guide for the Brazilian Population. Epidemiol Serv Saude. 2025;34:e20240369.

**DOI:** 10.1590/S2237-96222025v34e20240369.a

**Published:** 2025-05-12

**Authors:** Bárbara dos Santos Simões

**Affiliations:** Universidade José do Rasário Vellano

**Table:** 

Rating	Excellent	Good	Below Average	Poor
Interest	✓			
Quality		✓		
Originality		✓		
Overall		✓		

**Table:** 

Questionnaire	Yes	No	Not applicable
Does the manuscript contain new and significant information to justify publication?	✓		
Does the Abstract (Summary) clearly and accurately describe the content of the article?	✓		
Is the problem significant and concisely stated?	✓		
Are the methods described comprehensively?	✓		
Are the interpretations and conclusions justified by the results?	✓		
Is adequate reference made to other work in the field?	✓		

## Manuscript Structure:

Length of article is: Adequate

Number of tables is: Adequate

Number of figures is: Adequate

Please state any conflict(s) of interest that you have in relation to the review of this paper (state “none” if this is not applicable): None

## Comments

O artigo possui temática interessante e atual. Agora, com o lançamento da questnova, a análise sobre o consumo alimentar utilizando a Nova ficará mais adequando. Sucesso!

